# Molecular and cytogenetic analyses in *Geranium macrorrhizum* L. wild Italian plants

**DOI:** 10.1098/rsos.240035

**Published:** 2024-04-10

**Authors:** Irene Cardinali, Marilena Ceccarelli

**Affiliations:** ^1^ Department of Chemistry, Biology and Biotechnology, University of Perugia, Perugia 06123, Italy

**Keywords:** chromosome number, DNA barcoding, FISH, *Geranium macrorrhizum *L, phylogenetic analysis

## Abstract

*Geranium macrorrhizum* L. is a herbaceous species native to southern Europe and was introduced in central Europe and North America. It is also widely distributed in Italy. In this study, molecular and cytogenetic analyses were carried out on 22 wild plants, collected in central and southern Italy, compared with five cultivated plants, with the main purpose to identify those living near the Marmore waterfalls in central Italy, recently described as the new species *Geranium lucarinii*. Four barcoding markers (*rbcL*, *matK*, *trnH-psbA* intergenic spacer and internal transcribed spacer region) were sequenced and their variability among the plants was evaluated. Chromosome numbers were determined and 45S rDNA was physically mapped by fluorescence *in situ* hybridization. Moreover, genomic affinity between wild and cultivated plants was evaluated by genomic *in situ* hybridization. The results of this study supported that all the plants belong to *G. macrorrhizum*, including the Marmore population. Barcoding analyses showed a close similarity among the wild plants, and a differentiation, although not significant, between the wild plants on one hand and the cultivated plants on the other. Integrated studies focusing on morphological, genetic and ecological characterization of a larger number of wild populations would allow us to know the extent of the variability within the species.

## 1. Introduction

Accurate species delimitation is fundamental to biology. It has implications not only for a reliable evaluation of biodiversity but also for the use of the organisms at many levels, even for their conservation [1,2]. It is now widely accepted that alpha taxonomy, based on morphological characters, could not be sufficient to guarantee a reliable species description and delimitation. Rather, the integrated use of molecular, karyological and ecological data, along with quantitative morphology, is strongly recommended ([3–5] and references therein).

A rapid and reliable molecular method to identify a species is the DNA barcoding approach [6,7]. It is based on sequences from a few short, standardized, plastid DNA regions, tested for their universality and discrimination power. The two-marker combination of *rbcL* and *matK* is the core barcode in plants, usually used in conjunction with other markers, including nuclear ones (i.e. nuclear internal transcribed spacer region (ITS)) [8]. DNA barcoding has been largely used in basic and applied biodiversity research to discriminate between morphologically similar taxa, thus reducing the number of misidentifications, for cultivated flora analyses or to solve the doubtful status of some alien species ([9] and references therein, [10]).

*Geranium macrorrhizum* L. is a herbaceous perennial species with a robust and more or less horizontal rhizome. The aerial stem is erect, quite long, naked up to the inflorescence with zygomorphic flowers, pink or purplish in colour. Basal leaves are in persistent rosette, whereas cauline leaves are opposite, five-lobed (palmate), with glandular and eglandular hairs [11,12]. Essential oil containing the monoterpenoids geraniol and beta-citronellol, and several sesquiterpenes including germacrone, are extracted from the aerial parts of the plant to be used in aromatherapy and phytotherapy [13,14]. However, the species is mainly cultivated as an ornamental plant, with cultivars selected for flower colours from white through pink to magenta [12]. Regarding the chromosome number, two cytotypes corresponding to two ploidy levels, 2*n* = 2*x* = 46 and 2*n* = 4*x* = 92, were observed [15–20], although variable chromosome numbers in the range 87–93 were also reported [21].

*Geranium macrorrhizum* belongs to the subgenus *Robertium* (Picard) Rouy, section *Unguiculata* (Boiss.) Reiche, including, at present, only one other species, *Geranium dalmaticum* (Beck) Rech. f., with 2*n* = 2*x* = 46 [11,12,22–24]. A close morphological similarity exists between the two species, so that in the past *G. dalmaticum* had been considered a subspecies or a variety of *G. macrorrhizum* (cf. [11]). A third species, *G. kikianum* Kit Tan & Vold, endemic to the Taigetos Mountains (Peloponnese, Greece), tetraploid as *G. macrorrhizum*, was ascribed to this section [20], but considered a synonym of *G. macrorrhizum* after an in-depth revision [11].

*Geranium macrorrhizum* has a wide range of distribution, being native to southern Europe, from south-eastern France to Krym, and introduced in central Europe [11] and North America [25]. In Italy, the species is present in most of the northern and central regions, with few exceptions (Toscana, Marche), whereas it has been reported in only two southern regions (Molise and Campania). In Umbria (central Italy), it is found only near the Marmore waterfalls, where it was discovered in 1837 [26], observed again about 30 years later [27], and accidentally rediscovered during field research in 2016 [28]. Morphological analyses carried out on both living plants and herbarium samples highlighted that this population (hereinafter called Marmore population) diverged from *G. macrorrhizum* for three traits related to indumentum, leaves and calyx [29]. Notable differences also exist in the ecological niche (altitude and vegetation context). Indeed, almost all the Apennine and peninsular stations of *G. macrorrhizum* are located in the mountain belt on calcareous rocks and also in beech forests, or in orophilic screes, at over 1000 m.a.s.l., whereas the Marmore population lives on calcareous rocky slopes, at 190–250 m.a.s.l., in a Mediterranean vegetation context [29]. On these bases, the Marmore population was elevated to the rank of species, called *Geranium lucarinii* Venanzoni & Wagens. [29]. The new species has a restricted and punctiform distribution. It is represented only by that small population in an anthropically disturbed environment, for which it has been considered a critically endangered species, according to the International Union for Conservation of Nature [30] criteria [29]. However, in a recent monography about the *Geranium* genus, the name *G. lucarinii* (Venanzoni & Wagens.) is cited as a synonym of *G. macrorrhizum* L. [23].

With the aim of testing the identity of the plants collected at Marmore waterfalls and to shed light on the relationships between the putative new species *G. lucarinii* and *G. macrorrhizum*, DNA barcoding was carried out on Marmore plants, compared with *G. macrorrhizum* wild plants from central and southern Italy and cultivated plants. The use of plastid barcode markers proves to be particularly appropriate for studying relationships between *G. lucarinii* and *G. macrorrhizum,* owing to the large structural variation and high rates and patterns of nucleotide substitutions observed in plastomes of different *Geranium* species shown [31]. In addition to the core barcode markers *rbcL* and *matK*, two supplementary markers, nuclear ITS and plastid intergenic spacer *trnH-psbA*, were used [6,8]. The latter has never been sequenced in *G. macrorrhizum* before, but it was used anyway because the high variability makes it a particularly suitable marker for discriminating between closely related species [8,32,33]. Furthermore, cytogenetic analysis was undertaken in order to know the chromosome number of the plants studied and to characterize their chromosome complement by means of fluorescence *in situ* hybridization (FISH) of 45S rDNA. Finally, cross-genomic *in situ* hybridization (GISH) experiments were carried out to assess the genomic affinity between Marmore plants and the cultivated ones. This method is commonly applied to reveal genomic similarity between closely related species based on the homology of the repetitive DNA sequences ([34] and references therein).

## 2. Material and methods

### 2.1. Plant material

**Figure 1.**
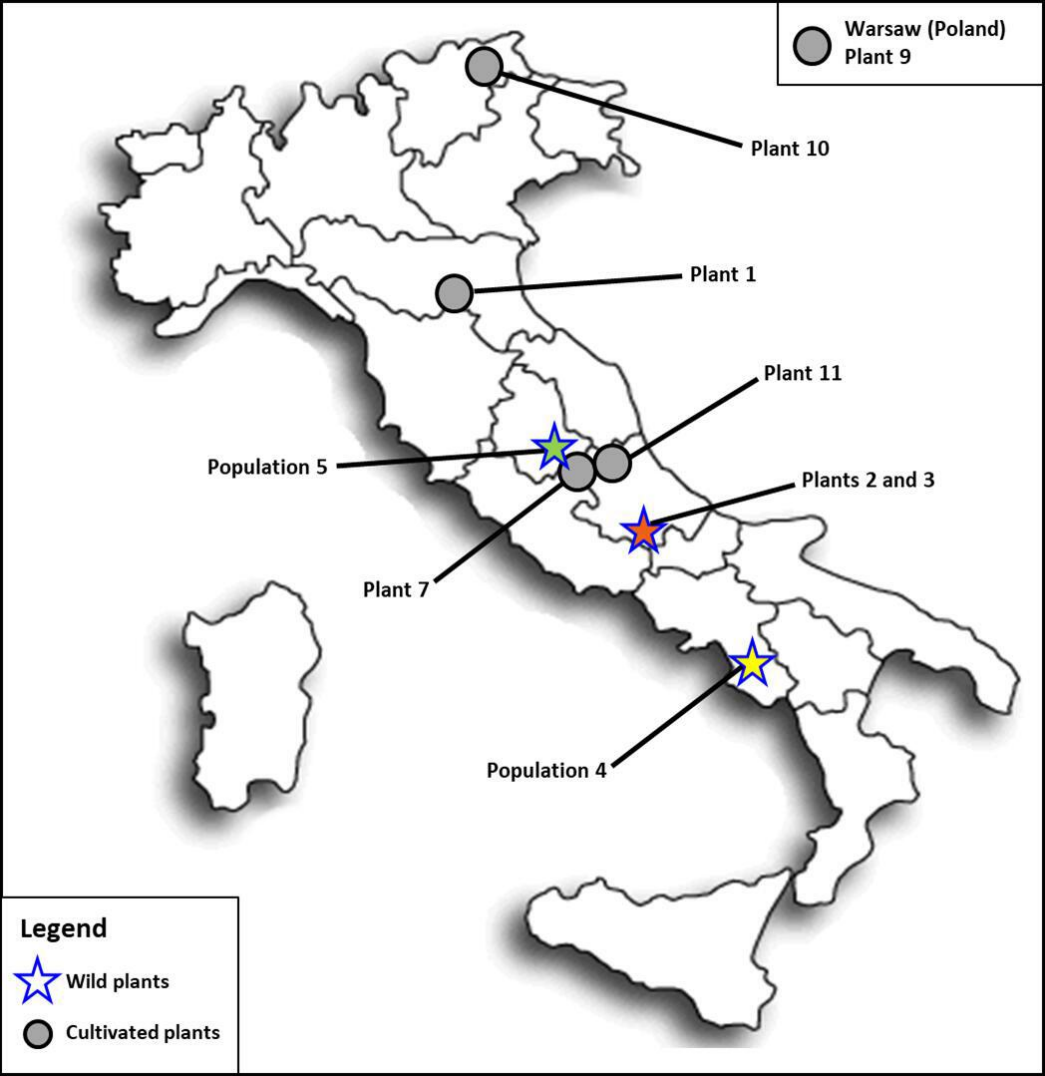
Sampling location of wild (stars) and cultivated (grey circles) plants in Italy. Plant 9 was collected in Warsaw (Poland), here indicated in a separate box. The provenance of wild plants is highlighted with different colours: green for Marmore waterfalls, orange for NPALM and yellow for Felitto (see table 1 for details).

Plants were collected in field inspections in central and southern Italy, at Marmore waterfalls and Felitto (Campania Region), respectively, by Prof. R. Venanzoni and Prof. R. P. Wagensommer in 2019 and 2020. Ten plants per population were sampled. As the species are rhizomatous, care was taken to collect plants at a suitable distance from each other. The plants from Felitto had been described as *G. macrorrhizum* [35,36]. Two plants of *G. macrorrhizum*, previously collected at the National Park of Abruzzo, Latium and Molise (NPALM; central Italy) and then transferred to the Botanical Garden of Camerino University (central Italy), were obtained by R. Venanzoni from this institution. Other *G. macrorrhizum* plants were obtained from Botanical or public gardens (table 1 and figure 1). All the plants were cultivated *ex situ* at the Department of Chemistry, Biology and Biotechnology of Perugia University and used for molecular and cytogenetic analyses.

**Table 1.** List of the plants studied, their status and geographical provenance.

sample	status	provenance
plant 1	cultivated	Bologna (Emilia-Romagna, northern Italy), Botanical Garden
plant 2	wild	National Park of Abruzzo, Latium and Molise (central Italy)
plant 3	wild	National Park of Abruzzo, Latium and Molise (central Italy)
plants from 4a to 4l (population 4)	wild	Felitto (southern Campania, Italy)
plants from 5a to 5n (population 5)	wild	Cascata delle Marmore (Marmore waterfalls, Umbria, central Italy)
plant 7	cultivated	Mount Terminillo (Latium, central Italy), public garden
plant 9	cultivated	Warsaw (Poland), public garden
plant 10	cultivated	Vipiteno (Trentino Alto-Adige, northern Italy), public garden
plant 11	cultivated	Campotosto (Abruzzo, central Italy), public garden

### 2.2. DNA extraction, amplification and sequencing

Total genomic DNA was isolated from fresh leaves using the DNeasy Plant Mini kit (Qiagen, Germany) according to the manufacturer’s instructions.

Three plastid markers (*rbcL*, *matK* and *trnH-psbA* intergenic spacer) and the nuclear ITS region (ITS1-5.8S-ITS2), were amplified in a 25 µl volume reaction containing 20 ng of DNA template, 1 µl of each primer (10 pmol µl^-1^) and 0.5 units of MyTaq HS polymerase (Bioline). Amplifications were performed on a thermal cycler 2720 (Applied Biosystems, Foster City, CA, USA). The primer pairs and cycling conditions are listed in the electronic supplementary material, table S1. Two *matK* primer pairs were used. First, the 390F + 1326R pair [37] failed amplification. The primer pair 3F + 1R KIM [38] produced multiple sequences. In order to obtain a single amplicon, the 1R KIM sequence was modified according to the complementary region on the *G. macrorrhizum matK s*equences found in the GenBank database. Amplified products were purified using the ExoSAP-IT® *Express* reagent (Thermo Fisher Scientific Inc.). Sequencing in both directions was performed by Eurofins Genomics service (Germany). Primers used for sequencing were the same as those for amplifications. Electropherograms quality was visually inspected. Sequences were manually edited and aligned using the ClustalW algorithm implemented in BioEdit 7.1.7 [39] with the default values. The sequences were compared with those available in GenBank (cf. electronic supplementary material, table S2) through a BLASTn search [40].

Newly determined sequences were deposited in GenBank (accession numbers OK299101 for *rbcL*, OM417815 for *trnH-psbA*, from OR656480 to OR656483 for ITS, OR668227 for *matK*). Only differing sequences for each locus have been deposited.

### 2.3. Phylogenetic analyses

The identification of variable and parsimony informative sites was carried out using MEGA 11 software [41]. MUSCLE [42] was used to align the sequences with the outgroup ones in MEGA 11. Genetic relationships among samples were inferred using both neighbour-joining (NJ) [43] and maximum likelihood (ML) methods. In NJ analyses, the genetic distances were computed using the Kimura 2-parameter (K2P) substitution model [44] for each locus and were given as units of the number of base substitutions per site. All ambiguous positions were removed for each sequence pair. Bootstrap analysis was done using 1000 replicates [45]. For ML analyses, the Tamura-Nei model was used [46]. Initial trees for the heuristic search were obtained automatically by applying NJ and BioNJ algorithms to a matrix of pairwise distances and then selecting the topology with superior log likelihood value. Branch lengths measure the number of substitutions per site. Bootstrap analysis was done using 1000 replicates. Three *Geranium* species whose ITS sequence showed the highest identity percentage (97.75–97.27%) with GenBank *G. macrorrhizum* sequence DQ525073 were added as outgroups (*Geranium dalmaticum* DQ525072, *Geranium lasiopus* Boiss. & Heldr. KX421242, and *Geranium glaberrimum* Boiss. & Heldr. KX421239). Two species for which both *rbcL* and *matK* sequences from the same origin were available, were chosen as outgroups (*Geranium lucidum* L. MK542503 and JN896161, and *Geranium robertianum* L. KP963378 and KY687141). Moreover, all the *rbcL* and *matK G. macrorrhizum* sequences available in GenBank were aligned to obtain those dendrograms (see accession numbers in dendrograms). NJ and ML trees were constructed both for each marker and for concatenated markers. In concatenated trees, sequences of *G. macrorrhizum* and outgroup species were obtained by the sum of three marker sequences probably deriving from different individuals. Genetic relationships among plants were also investigated through a median-joining network of haplotypes obtained by analysing the concatenated sequences. The network was constructed with the Network software v.10.2 (www.fluxus-engineering.com) by using the reduced median algorithm (*ρ* = 2). The term haplotype used here indicates the list of mutations found in the examined sequences in each sample, arbitrarily numbered for the analyses.

### 2.4. Cytogenetic analyses

Root apices were treated with ice-cold water for 24 h at 4°C, then transferred in 8-OH-quinoline (Sigma) 0.02 M for 5 h at room temperature and fixed in ethanol-acetic acid 3 : 1 (v/v). Fixed root tips were washed in an aqueous solution of 6 mM sodium citrate plus 4 mM citric acid, digested with a mixture of 10% pectinase (Sigma), 8% cellulose (Calbiochem) and 2% macerozyme (Serva) in citrate buffer pH 4.6 for 1 h at 37°C and squashed under a coverslip in a drop of 60% acetic acid. After removing coverslips by the solid CO_2_ method, slides were air-dried and used for FISH or GISH experiments. FISH was performed as described in Mascagni *et al*. [47]. The wheat probe pTa71, containing 18S-5.8S-26S rDNA [48] was used. The DNA of nuclei and chromosomes was denatured in a thermal cycler for 6 min at 70°C and the preparations were incubated overnight at 37°C with 2 ng µl^-1^ of heat-denatured DNA probe which had been labelled with digoxigenin-11-dUTP (Roche) or biotin-16-dUTP (Roche) by nick translation. Detection of the digoxigenin or biotin at the hybridization sites was carried out using anti-digoxigenin conjugated with fluorescein isothiocyanate (FITC; Roche) or streptavidin conjugated with Cy3 (Cyanine 3; Sigma), respectively. The preparations were then counterstained with a 2% 4,6-diamidino-2-phenylindole (DAPI) solution in McIlvaine buffer pH 7, mounted in antifade solution (AFI; Citifluor) and analysed with a fluorescence microscope (DMRB, Leica). Images were captured with a digital camera (ILCE-7, Sony) and optimized using Adobe Photoshop 5.0.

The same slides were used for chromosome counting. At least 10 DAPI-stained metaphases per plant were analysed.

For GISH experiments (self-GISH and cross-GISH), total genomic DNAs extracted from leaves were used as probes after labelling with biotin-11-dUTP by nick translation following the producer’s protocol (BioNick Labeling System, Invitrogen). The GISH procedure was similar to the FISH protocol with the exception of the probe concentration, which was 5 ng µl^-1^. These experiments were replicated three times.

## 3. Results

Sequences of different lengths were obtained for each marker (table 2). The *rbcL* sequences used for subsequent analyses were longer than the gene fragment, 599 bp in length, considering the barcode region by Hollingsworth *et al*. [8]. *matk* locus proved to be difficult to amplify. The mainly used primer combinations failed in amplification, therefore a specific reverse primer was designed and used in combination with primer 3F KIM (electronic supplementary material, table S1). Also in this case, the primer pair used produced sequences slightly differing in length from the gene barcode region [8]. BLASTn analysis showed that all the *rbcL* and *matK* sequences, either from wild or cultivated plants, were identical to those of *G. macrorrhizum*. Indeed, the identity percentage was in the range 99.86–100.00 and 99.87–100.00 for *rbcL* and *matK* sequences, respectively (electronic supplementary material, table S2). The range was slightly wider for ITS sequences (98.55–100.00; electronic supplementary material, table S2). Regarding the intergenic spacer *trnH-psbA*, sequences 339–346 bp in length were obtained. They resulted monomorphic in nucleotide composition and showed the highest identity percentage (95%) with *Geranium maderense* Yeo sequence (electronic supplementary material, table S2). Their alignment with the plastome of *G. maderense* showed that the intergenic spacer *trnH-psbA* in the analysed plants is 288 bp long and has a G + C content of 36.1%.

**Table 2.** Range of the amplicon sequences for each marker and some characteristics of the fragments aligned for NJ and ML trees.

	*rbcL*	*matK*	ITS	*trnH-psbA*
sequences length (bp)	692–703	822–837	677–709	339–346
aligned length for NJ and ML trees (bp)	692	787	623	Not aligned
no. of variable sites among aligned sequences	1	1	4	0
no. of parsimony informative sites	1	1	4	0

### 3.1. Phylogenetic analyses

**Figure 2.**
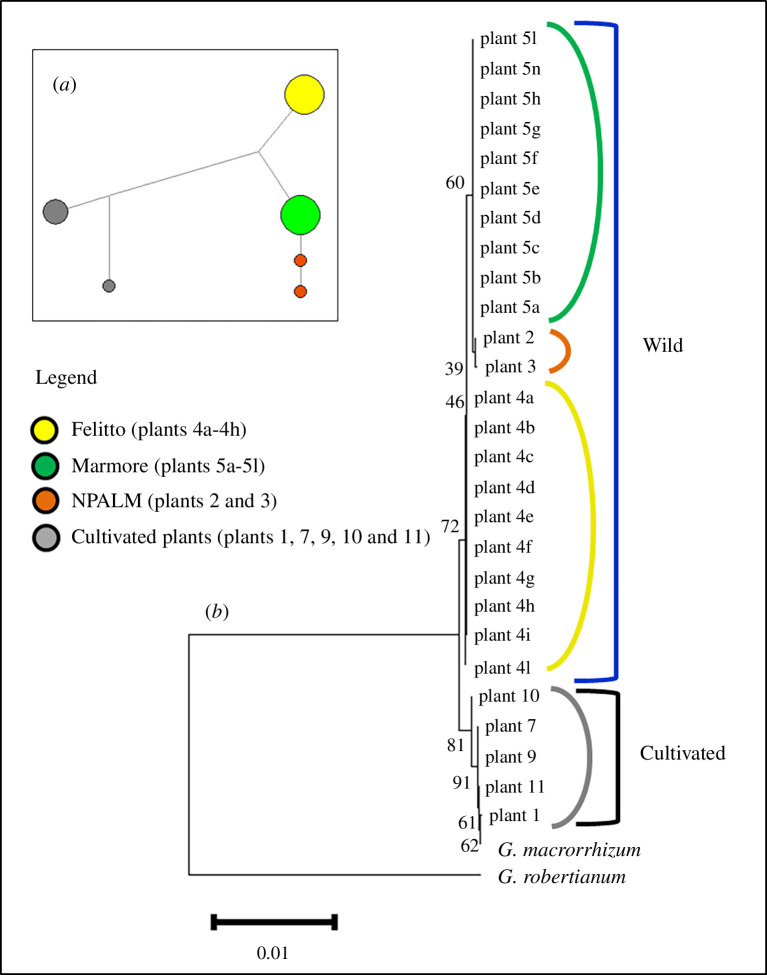
Reduced median network (*a*) of wild and cultivated plants based on their concatenated sequences (ITS +*rbcL +* matK). Circles represent the haplotypes and are proportional to the observed frequency in the analysed plants (electronic supplementary material, table S3), while colours (as in figure 1) indicate their geographical provenance. NPALM, National Park of Abruzzo, Latium and Molise. NJ tree (*b*) encompassing the concatenated sequences (ITS + *rbcL +* matK) from this study and those available from GenBank, obtained by concatenating ITS, *rbcl* and *matK* sequences from *G. macrorrhizum* (DQ525073, KP963387 and KY687134, respectively) and *G. robertianum* (DQ525071, KP963378 and KY687141, respectively). Plants numbering as in table 1. Bootstrap values are indicated next to the branches.

The network analysis, constructed with the reduced median method, was applied to analyse the genetic relationships among the plants. To this purpose, sequences were aligned and trimmed for each marker, and concatenated sequences were used. Six haplotypes were found, four within the wild plants and two within the cultivated ones (figure 2*a*). Population 4 (Felitto, Campania) was homogeneous such as population 5 (Marmore). The two plants from NPALM (plants 2 and 3) showed unique haplotypes, as well as the cultivated plant 10. All the other cultivated samples shared the same haplotype. Within *rbcL* sequences, only a single nucleotide polymorphism (SNP) was found, consisting of a transversion A/C (nucleotide position 472 in the electronic supplementary material, table S3) which distinguishes the plants from Marmore and NPALM from all the others. Only one SNP, a transition C/T (nucleotide position 149 in the electronic supplementary material, table S3), was also observed among *matK* sequences, distinguishing all the wild plants from the cultivated ones. Instead, 13 polymorphic sites were observed among ITS sequences. All of them were located in the sequenced portions of the intergenic spacers ITS1 and ITS2. Four polymorphic sites distinguished cultivated plants from the wild ones. Several polymorphic sites were heteroplasmic nucleotides in the *G. macrorrhizum* sequence available in GenBank (DQ525073) and only in those of cultivated plants. Heteroplasmy was shown at two sites in wild population 4 and one site in plant 2 from NPALM (electronic supplementary material, table S3).

Bearing in mind the need to clarify the taxonomical placement of the Marmore population, the genetic relationships among plants were further investigated by NJ and ML methods, including sequences of *G. macrorrhizum* and outgroup species retrieved from GenBank. Some characteristics of the trimmed aligned fragments are shown in table 2. The only polymorphism detected among *rbcL* sequences (see above) was responsible for a weakly supported but clear differentiation among wild plants (electronic supplementary material, figure S1). Instead, all the wild plants were included in the main branch of the *matK* tree, harbouring a weakly supported sub-cluster including the cultivated ones and two samples of *G. macrorrhizum* from GenBank (electronic supplementary material, figure S2). The concatenated *rbcL+ matK* tree*,* based on a total of 1479 bp*,* showed a highly supported main branch harbouring all the analysed plants. The cultivated plants closely clustered with *G. macrorrhizum* samples, whereas a further differentiation emerged among the wild ones (electronic supplementary material, figure S3). The ITS tree harboured two main clades, the first including all the wild plants, while the second comprising the cultivated plants (electronic supplementary material, figure S4). Some variability can be observed within each cluster. The two plants from NPALM formed a sub-cluster, whereas population 4 was slightly differentiated from population 5. Among the cultivated plants, sample 10 turned out to be more similar to *G. dalmaticum* than to *G. macrorrhizum*. The concatenated tree *rbcL + matK* + ITS, for a total of 2101 bp (figure 2*b*) included only *G. robertianum* as an outgroup because the sequences of all the three markers were available in GenBank only for this species. The tree highlighted the differences between the two groups of cultivated and wild plants, already observed in figure 2*a*. The wild plants were in turn grouped into three sub-clusters corresponding to their geographical provenance. Although ML trees showed the same NJ topology, the ML concatenated tree is reported in the electronic supplementary material, figure S5.

### 3.2. Cytogenetic analysis

Chromosome counts on the DAPI stained metaphases showed that the somatic chromosome number in all the wild plants, including those from Marmore, was 2*n* = 92, whereas in the cultivated plants it was 2*n* = 46, with the exception of plant 10, showing 2*n* = 92.

**Figure 3.**
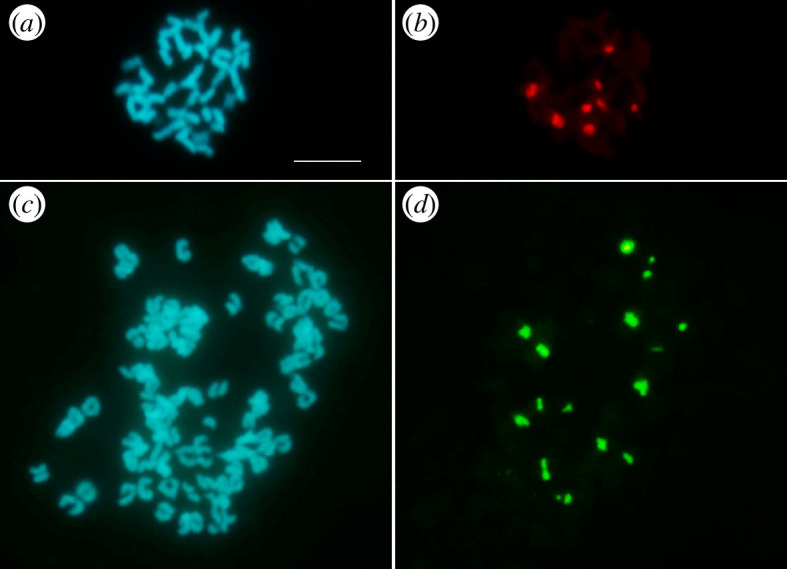
Metaphase plates of cultivated plant 1 (*a*, *b*) and Marmore plant 5e (*c*, *d*); after DAPI staining (*a*, *c*) and hybridization with pTa71 ((*b*) Cy3; (*d*) fluorescein). Bar represents 10 µm.

Owing to the small chromosome size, it was difficult to arrange the karyotype. In order to establish at least the number of chromosome pairs carrying ribosomal DNA, FISH was carried out using pTa71 as a probe. Eight hybridization signals related to 45S rDNA were counted on metaphase plates of cultivated plants (figure 3*a,b*), whereas a maximum of 16 signals were observed on metaphase plates of wild plants, comprising those collected at Marmore waterfalls (figure 3*c,d*).

**Figure 4.**
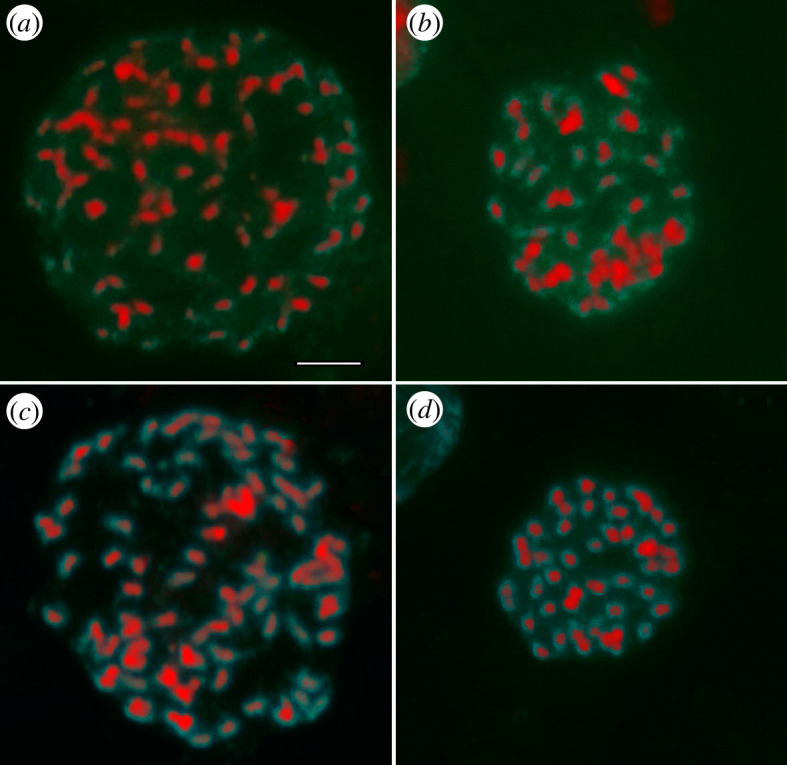
Pro-metaphases of Marmore plant 5e (*a*) and cultivated plant 11 (*b*) after hybridization with their own labelled genomic DNA (self-GISH). Pro-metaphase of Marmore plant 5e hybridized with biotin-labelled genomic DNA from cultivated plant 11 (*c*) and pro-metaphase of cultivated plant 11 hybridized with biotin-labelled genomic DNA from Marmore plant 5e (*d*). Labelled genomic DNAs were revealed by Cy3. Bar represents 10 µm.

To evaluate the genome affinity between Marmore plants and *G. macrorrhizum*, GISH experiments were carried out by probing the genomic DNA of Marmore plants on chromosomes of cultivated plants and vice versa (figure 4). Preliminary experiments in which the labelled DNA of Marmore plants or cultivated plants was hybridized to its own chromosomes (self-GISH) were performed to better evaluate, by comparison, the results of cross-GISH. Thus, after self-GISH, fluorescent signals, although of different intensity, were observed on each chromosome of the complement in both wild and cultivated plants (figure 4*a,b*). Low-intensity signals were easily recognized at the centromeric and pericentromeric regions, showing a hybridization pattern typical of satellite DNA. The same hybridization pattern was observed after cross-GISH (figure 4*c,d*).

## 4. Discussion

In this study, DNA barcoding was applied to test the identity of plants living near the Marmore waterfalls in central Italy, considered a new species, *G. lucarinii* [29]. The plants are morphologically so similar to *G. macrorrhizum* [29] that recently *G. lucarinii* has been considered its synonym [23].

All the sequences of markers *rbcL*, *matK* and ITS showed identity percentages equal or close to 100% with those of *G. macrorrhizum*. The greatest variability was observed among ITS sequences, as expected owing to its nature of a bi-parentally inherited marker. The minimum value of the range (98.55%) was higher than the identity percentage (97.76%) between ITS of *G. macrorrhizum* DQ525073 and that of the closely related species *G. dalmaticum* DQ525072, confirming that our ITS sequences correspond to *G. macrorrhizum*.

*trnH-psbA* intergenic spacer did not contribute to species identification because the GenBank database was missing the reference sequence for *G. macrorrhizum*. However, it proved likewise useful. Indeed, any *trnH-psbA* sequence variation was observed among the plants here analysed. This sequence monomorphism, unusual for the marker, supports the fact that all the analysed plants seem to belong to the same species.

The distribution of genetic variability in barcoding sequences suggested some differentiation within the plants studied (figure 2). The cultivated plants closely clustered with *G. macrorrhizum*, whereas wild plants were clearly grouped into three sub-clusters corresponding to their geographical origin. The plants from the NPALM were more closely related to the Marmore population than to population 4 from Campania (Felitto). Interestingly, the two samples from the National Park are the same plants used to morphologically compare plants from Marmore, later considered a new species [29]. Despite this clustering, it is clear that genetic variation between cultivated and wild plants does not support the existence of two different species.

This finding is also confirmed by the cytogenetic analyses. Two cytotypes, diploid and tetraploid, were detected in this study. Ninety-two chromosomes, corresponding to the tetraploid level, were counted in Marmore plants, as well as in the other wild plants studied, versus the 46 chromosomes counted in almost all the cultivated plants, with the exception of plant 10. However, the different chromosome numbers cannot be considered a discriminating factor, because the existence of diploid and tetraploid plants with *n* = 23 has long been known in *G. macrorrhizum* [15–21]. Recently, plants with a genome size corresponding to the hexaploid level were found in Croatia [49]. The occurrence of different ploidy levels in the same species, owing to endopolyploidy, is not exclusive to *G. macrorrhizum*. Rather, it is common to many taxa of the *Geranium* genus [50].

Our FISH analyses confirmed that wild plants have a doubled chromosome number compared with the cultivated ones (figure 3). The number of 45S rDNA signals was in agreement with the ploidy level, unlike what occurs in many species in which a reduction in the number of ribosomal DNA sites per monoploid genome is observed following polyploidization [51]. FISH also showed that the number of chromosome pairs carrying ribosomal DNA is higher than that previously observed in karyotype analyses carried out in different *G. macrorrhizum* Bulgarian populations with 2*n* = 46 [18]. Indeed, eight hybridization signals, corresponding to four chromosome pairs, were observed in our cultivated plants with 46 chromosomes, whereas only two or three nucleolar chromosome pairs were found by Petrova & Stanimirova [18].

Genomic affinity between Marmore plants and *G. macrorrhizum* was cytologically investigated by GISH (figure 4). Repetitive DNA sequences (satellite DNA) are mainly involved in the hybridization reaction. The method provides a powerful tool to study their distribution pattern along chromosomes, especially in species for which there is a lack of genome information [52,53]. Since most satellite DNA sequences are fast evolving in structure, redundancy and localization even within the same species [54,55], their detection through GISH could give information about the relationship between related species. The comparison of hybridization patterns after self-GISH and cross-GISH in our material showed the homology of the repeated sequences between Marmore plants and the cultivated ones.

Thus, at present, our molecular and cytogenetic data support the presence of only species *G. macrorrhizum* L. in central and southern Italy, confirming that the name *G. lucarinii* Venanzoni & Wagens. becomes a synonym. Evidently, differences in the few morphological features discriminating the two species have been considered by Aedo [23] as a part of the morphological variability characterizing *G. macrorrhizum*. Further traits distinguishing *G. lucarinii* from *G. macrorrhizum* were the flowering period and the habitat, in terms of vegetation context and altitudinal range of distribution [29]. The latter (190–250 m.a.s.l.) is partly overlapping with that of *G. macrorrhizum* (50–2800 m.a.s.l.). In addition, one of the most southern *G. macrorrhizum* Italian stations, Felitto (population 4 in this study), is also located at a low altitude (200–290 m.a.s.l. [35,36]). Differences in flowering period and morphology observed between Marmore plants and *G. macrorrhizum* could be owing to adaptation to environmental factors. For example, leaf traits previously used to delimit species in *Rhodiola* sect. *Trifida* were then found to be strongly influenced by climatic variables related to rainfall [2]. A comparative climatic niche analysis could help to clarify this issue.

This study is, to our knowledge, the first report on molecular and cytogenetic characterization of *G. macrorrhizum* Italian populations. The topology of concatenated trees (figure 2*b*; electronic supplementary material, figure S5) suggests that *G. macrorrhizum* wild populations in central and southern Italy form a genetically fairly homogeneous group, well separated from the cultivated plants. The origin of cultivated plants here studied is not well known, just as there is not enough information on the status, if cultivated or wild, of *G. macrorrhizum* plants whose sequences were retrieved from GenBank. Beyond this, it is significant that the cultivated plants cluster together and with known *G. macrorrhizum*, whether it be cultivated or not, whereas the wild plants form a distinct cluster. Work is in progress to deepen morphological and genetic studies, extending them to a greater number of wild populations, to estimate the degree of the variability within the species. For the same purpose, the role played by the geographical distribution of the populations, their spatial isolation and consequent gene flow, as well as ecological specialization, will be evaluated. Such an integrative approach is fundamental to define different aspects of the speciation process and to delimit evolutionary distinct lineages [3,4].

## Data Availability

Our paper contains new sequences deposited in GenBank, and whose accession numbers are listed in the main text. Electronic supplementary material is available online [56].

## References

[1] Garnett ST , Christidis L . 2017 Taxonomy anarchy hampers conservation. Nature **546** , 25–27. (10.1038/546025a) 28569833

[2] Li Y , Wen J , Ren Y , Zhang J . 2019 From seven to three: integrative species delimitation supports major reduction in species number in Rhodiola section Trifida (Crassulaceae) on the Qinghai‐Tibetan Plateau. Taxon **68** , 268–279. (10.1002/tax.12052)

[3] De Queiroz K . 2007 Species concepts and species delimitation. Syst. Biol. **56** , 879–886. (10.1080/10635150701701083) 18027281

[4] Dejaco T , Gassner M , Arthofer W , Schlick-Steiner BC , Steiner FM . 2016 Taxonomist’s nightmare … evolutionist’s delight: an integrative approach resolves species limits in jumping bristletails despite widespread hybridization and parthenogenesis. Syst. Biol. **65** , 947–974. (10.1093/sysbio/syw003) 26869489 PMC5066060

[5] Tiburtini M *et al* . 2022 Integrative taxonomy of Armeria arenaria (Plumbaginaceae), with a special focus on the putative subspecies endemic to the Apennines. Biology (Basel) **11** , 1060. (10.3390/biology11071060) 36101438 PMC9312046

[6] Hollingsworth PM . 2009 A DNA barcode for land plants. Proc. Natl Acad. Sci. USA **106** , 12794–12797. (10.1073/pnas.0905845106) 19666622 PMC2722355

[7] Hebert PDN , Cywinska A , Ball SL , deWaard JR . 2003 Biological identifications through DNA barcodes. Proc. R. Soc. B **270** , 313–321. (10.1098/rspb.2002.2218) PMC1691236 12614582

[8] Hollingsworth PM , Graham SW , Little DP . 2011 Choosing and using a plant DNA barcode. PLoS One **6** , e19254. (10.1371/journal.pone.0019254) 21637336 PMC3102656

[9] De Castro O , Del Guacchio E , Di Iorio E , Di Maio A , Di Martino L , Bartolucci F , Conti F . 2020 Barcoding helps threatened species: the case of Iris marsica (Iridaceae) from the protected areas of the Abruzzo (Central Italy). Plant Biosyst. **154** , 961–972. (10.1080/11263504.2020.1762786)

[10] Koblmüller S . 2023 DNA barcodes for evolution and biodiversity. Diversity **15** , 1003. (10.3390/d15091003)

[11] Aedo C . 2017 Taxonomic revision of Geranium Sect. Ruberta and Unguiculata (Geraniaceae). Ann. Mo. Bot. Gard. **102** , 409–465. (10.3417/D-16-00016A)

[12] Yeo PF . 2004 The morphology and affinities of Geranium sections Lucida and Unguiculata. Bot. J. Linn. Soc. **144** , 409–429. (10.1111/j.1095-8339.2003.00258.x)

[13] Harborne JB , Williams CA . 2002 Phytochemistry of the genus *Geranium* . In Geranium and Pelargonium. The genera Geranium and Pelargonium (ed. M Lis-Balchin ), pp. 20–29, London, UK: Taylor & Francis.

[14] Stoeva T . 2002 Cultivation and harvesting of *Geranium macrorrhizum* and *Geranium sanguineum* for medicinal use in Bulgaria. In Geranium and Pelargonium (ed. M Lis-Balchin ), pp. 30–35, London, UK: Taylor & Francis.

[15] Baltisberger M . 1991 Cytological investigations of some Greek plants. Fl. Medit **1** , 157–173.

[16] Baltisberger M Baltisberger E . 1995 Cytological data of Albanian plants. Candollea **50** , 457–493. (10.5169/seals-879478)

[17] Gauger W . 1937 Ergebnisse einer zytologischen Untersuchung der Familie der Geraniaceae. I. Planta **26** , 529–531. (10.1007/BF01914322)

[18] Petrova A Stanimirova P . 2002 In Mediterranean Chromosome numbers reports - 12 Fl Medit 12. [Reports 1288-1294]. (eds G Kamari , C Blanché , F Garbari ), pp. 443–486. Palermo, Italy: International Foundation pro Herbario Mediterraneo.

[19] Strid A , Franzén R . 1981 Chromosome number reports Lxxiii. In Taxon (ed. A Löve ), pp. 829–861, vol. **30** . Wiley Online Library. (10.1002/j.1996-8175.1981.tb04309.x)

[20] Tan K , Siljak‐Yakovlev S , Vold G . 2011 Geranium kikianum sp. nov. (Geraniaceae) from the southern Peloponnese, Greece. Nord. J. Bot. **29** , 1–5. (10.1111/j.1756-1051.2010.01028.x)

[21] Van Loon JC . 1984 Chromosome numbers in Geranium from Europe. I. The perennial species. Proc. Kon. Ned. Akad. Wetensch. Ser. C **87** , 263–277.

[22] Aedo C . 2018 Ajustes nomenclaturales en Geranium L. (Geraniaceae). Acta Bot. Malacit. **42** , 297–298. (10.24310/abm.v42i2.2750)

[23] Aedo C . 2023 A monograph of the genus Geranium L. Madrid, Spain: Consejo Superior de Investigaciones Cientificas (CSIC).

[24] Baltisberger M . 1984 Zytologische Untersuchungen an einigen Pflanzen aus Albanien. Ber Geobot Inst ETH, Stiftung Rubel, Zürich **51** , 63–77.

[25] Hawke R . 2004 Plant evaluation notes. Hardy geraniums for northern gardens. Chicago, IL: Chicago Botanic Garden.

[26] Sanguinetti P , Sebastiani A . 1837 Centuriae tres. Prodromo florae Romanae addendae. Romae: Ex Typographia Contedini. See https://www.biodiversitylibrary.org/bibliography/6725

[27] Fiorini Mazzanti E . 1869 Cenno sulla vegetazione della caduta delle Marmore in una rapida escursione di luglio. Atti Dell’Accademia Pontificia de’ Nuovi Lincei **22** , 143–144.

[28] Venanzoni R . 2017 Il Geranio Odoroso delle Marmore. In Il Giardino Botanico della Cascata delle Marmore – Biodiversità al lavoro (ed. R Venanzoni ), Terni, Italy: Comune di Terni, Fondazione Cassa di Risparmio di Terni e Narni.

[29] Wagensommer RP , Venanzoni R . 2021 Geranium lucarinii sp. nov. and re-evaluation of G. kikianum (Geraniaceae). Phytotaxa **489** , 252–262. (10.11646/phytotaxa.489.3.2)

[30] IUCN . 2019 Guidelines for using the IUCN Red List categories and criteria. Version 14. Prepared by the Standards and Petitions Committee, Cambridge UK. See https://www.iucnredlist.org/resources/redlistguidelines (accessed 18 January 2021).

[31] Park S , Ruhlman TA , Weng ML , Hajrah NH , Sabir JSM , Jansen RK . 2017 Contrasting patterns of nucleotide substitution rates provide insight into dynamic evolution of plastid and mitochondrial genomes of Geranium. Genome Biol. Evol. **9** , 1766–1780. (10.1093/gbe/evx124) 28854633 PMC5570028

[32] Federici S , Galimberti A , Bartolucci F , Bruni I , De Mattia F , Cortis P , Labra M . 2013 DNA barcoding to analyse taxonomically complex groups in plants: the case of Thymus (Lamiaceae): DNA barcoding of genus Thymus. Bot. J. Linn. Soc. **171** , 687–699. (10.1111/boj.12034)

[33] Kress WJ , Erickson DL . 2007 A two-locus global DNA barcode for land plants: the coding rbcL gene complements the non-coding trnH-psbA spacer region. PLoS ONE **2** , e508. (10.1371/journal.pone.0000508) 17551588 PMC1876818

[34] Falistocco E . 2019 Chromosome investigations in annual Medicago species (Fabaceae) with emphasis on the origin of the polyploid Medicago rugosa and M. scutellata. Plant Biosyst. **153** , 235–241. (10.1080/11263504.2018.1462864)

[35] Del Guacchio E . 2002 Note floristiche per la Campania. Delpinoa **44** , 75–80.

[36] Salerno G . 2004 Segnalazioni floristiche Italiane: 1127. Informatore Botanico Italiano **36** , 89.

[37] Cuénoud P , Savolainen V , Chatrou LW , Powell M , Grayer RJ , Chase MW . 2002 Molecular phylogenetics of Caryophyllales based on nuclear 18S rDNA and plastid rbcL, atpB and matK DNA sequences. Am. J. Bot. **89** , 132–144. (10.3732/ajb.89.1.132) 21669721

[38] Costion C , Ford A , Cross H , Crayn D , Harrington M , Lowe A . 2011 Plant DNA barcodes can accurately estimate species richness in poorly known floras. PLoS ONE **6** , e26841. (10.1371/journal.pone.0026841) 22096501 PMC3214028

[39] Hall T . 1999 BioEdit: a user-friendly biological sequence alignment editor and analysis program for windows 95/98/NT. Nucleic Acids Sympos. Ser. **41** , 95–98.

[40] Zhang Z , Schwartz S , Wagner L , Miller W . 2000 A greedy algorithm for aligning DNA sequences. J. Comput. Biol. **7** , 203–214. (10.1089/10665270050081478) 10890397

[41] Tamura K , Stecher G , Kumar S . 2021 MEGA11: molecular evolutionary genetics analysis, version 11. Mol. Biol. Evol. **38** , 3022–3027. (10.1093/molbev/msab120) 33892491 PMC8233496

[42] Edgar RC . 2004 MUSCLE: multiple sequence alignment with high accuracy and high throughput. Nucleic Acids Res. **32** , 1792–1797. (10.1093/nar/gkh340) 15034147 PMC390337

[43] Saitou N , Nei M . 1987 The neighbor-joining method: a new method for reconstructing phylogenetic trees. Mol. Biol. Evol. **4** , 406–425. (10.1093/oxfordjournals.molbev.a040454) 3447015

[44] Kimura M . 1980 A simple method for estimating evolutionary rates of base substitutions through comparative studies of nucleotide sequences. J. Mol. Evol. **16** , 111–120. (10.1007/BF01731581) 7463489

[45] Felsenstein J . 1985 Confidence limits on phylogenies: an approach using the bootstrap. Evolution **39** , 783–791. (10.1111/j.1558-5646.1985.tb00420.x) 28561359

[46] Tamura K , Nei M . 1993 Estimation of the number of nucleotide substitutions in the control region of mitochondrial DNA in humans and chimpanzees. Mol. Biol. Evol. **10** , 512–526. (10.1093/oxfordjournals.molbev.a040023) 8336541

[47] Mascagni F , Barghini E , Ceccarelli M , Baldoni L , Trapero C , Díez CM , Natali L , Cavallini A , Giordani T . 2022 The singular evolution of Olea genome structure. Front. Plant Sci. **13** , 869048. (10.3389/fpls.2022.869048) 35432417 PMC9009077

[48] Gerlach WL , Bedbrook JR . 1979 Cloning and characterization of ribosomal RNA genes from wheat and barley. Nucleic Acids Res. **7** , 1869–1885. (10.1093/nar/7.7.1869) 537913 PMC342353

[49] Ćavar Zeljković S , Siljak-Yakovlev S , Tan K , Maksimović M . 2020 Chemical composition and antioxidant activity of Geranium macrorrhizum in relation to ploidy level and environmental conditions. Plant Syst. Evol. **306** , 18. (10.1007/s00606-020-01649-9)

[50] Petrova A , Stanimirova P . 2003 Karyological study of some Geranium (Geraniaceae) species growing in Bulgaria. Bocconea **16** , 675–682.

[51] Garcia S , Kovařík A , Leitch AR , Garnatje T . 2017 Cytogenetic features of rRNA genes across land plants: analysis of the Plant rDNA database. Plant J. **89** , 1020–1030. (10.1111/tpj.13442) 27943584

[52] Falistocco E . 2023 Comparative cytogenetic study of Plantago species. Plant Biosyst. **157** , 1078–1084. (10.1080/11263504.2023.2242335)

[53] Zhang Y , Cheng C , Li J , Yang S , Wang Y , Li Z , Chen J , Lou Q . 2015 Chromosomal structures and repetitive sequences divergence in Cucumis species revealed by comparative cytogenetic mapping. BMC Genomics **16** , 730. (10.1186/s12864-015-1877-6) 26407707 PMC4583154

[54] Garrido-Ramos MA . 2017 Satellite DNA: an evolving topic. Genes **8** , 230. (10.3390/genes8090230) 28926993 PMC5615363

[55] Thakur J , Packiaraj J , Henikoff S . 2021 Sequence, chromatin and evolution of satellite DNA. Int. J. Mol. Sci. **22** , 4309. (10.3390/ijms22094309) 33919233 PMC8122249

[56] Cardinali I , Ceccarelli M . 2024. Data from: Molecular and cytogenetic analyses in Geranium macrorrhizum L. wild Italian plants. FigShare. (10.6084/m9.figshare.c.7123859) PMC11004676 38601032

